# The complete mitogenome and phylogenetic analysis of *Thalassa montezumae* Mulsant, 1850 (Coleoptera: coccinellidae)

**DOI:** 10.1080/23802359.2024.2412230

**Published:** 2024-10-07

**Authors:** Immacolata Iovinella, Lucrezia Giovannini, Giuseppe Mazza, Bryan Naqqi Manco, Agostino Strangi

**Affiliations:** a CREA–Research Centre for Plant Protection and Certification, Florence, Italy; b Department of Environmental and Coastal Resources, National Environmental Centre, Providenciales, Turks, Caicos Islands

**Keywords:** *Toumeyella* natural enemies, biological control, complete mitochondrial genome, lady beetle, scale insect predator

## Abstract

*Thalassa montezumae* Mulsant is a coccinellid species recently discovered as predator of the pine tortoise scale *Toumeyella parvicornis.* In this study, the complete mitochondrial genome of *T. montezumae* collected in Turks and Caicos Islands in 2023 was sequenced using next-generation sequencing techniques. The circular mitochondrial genome is 16,981 bp long and contains 13 protein coding genes, 22 transfer RNA, and 2 rRNA genes. Gene order is identical to that of other Coccinellidae. Phylogenetic analysis confirms structure of Coccinellidae families and tribes.

## Introduction

Coccinellidae, commonly named ‘ladybirds’, ‘ladybeetles’ or ‘ladybugs’, is a widespread family of insects characterized by hemispherical body shape, and colored elytrae that completely cover wings. Several species such as *Adalia bipunctata* (Linnaeus, 1758), *Rodolia cardinalis* Mulsant, 1850, *Exochomus quadripustulatus* (Linnaeus, 1758), and many others are commonly used in organic crop farming and routinely sold as biocontrol agents by several companies (Kairo et al. 2013; Rondoni et al. 2021). However, their impact on the ecological equilibrium should be carefully assessed in classical biocontrol programs to avoid adverse environmental consequences, as happened for Arlequin lady beetle *Harmonia axyridis* (Pallas, 1773) (Brown et al. 2011).

**Figure 1.**
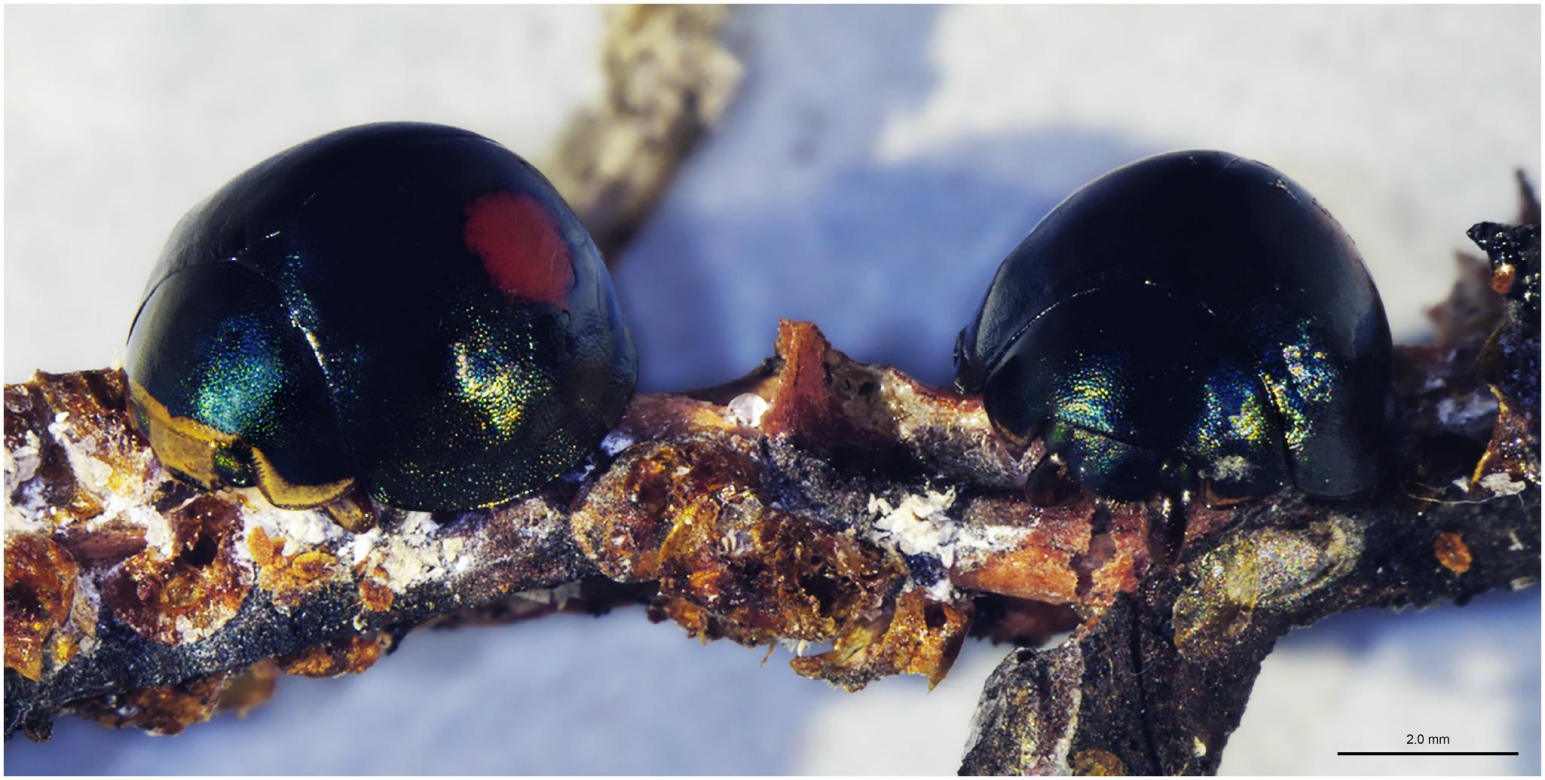
*Thalassa montezumae* male (on the left) and female (on the right). Picture taken with SMZ-25 stereomicroscope (NIKON) by Luca Madonni.

*Thalassa montezumae* Mulsant, 1850 (Figure 1 taken by Luca Madonni) is a coccinellid species native to Central America and southern part of the United States and the identification of this species is based solely on morphological characters (Gordon 1985; Milléo et al. 2004). No DNA sequences are available in public databases for any species belonging to the genus *Thalassa* Mulsant, 1850 and only few studies have been conducted on *T. montezumae* (Barahona et al. 2018; Francis et al. 2022). Recently, this species was discovered for the first time in the Turks and Caicos Islands as a predator of the invasive pest of *Toumeyella parvicornis* (Cockerell, 1897) (Giovannini et al. 2024). *Toumeyella parvicornis* has led to the near extinction of the Caribbean pine in the Turks and Caicos Islands and poses a serious threat to pine trees in other countries where it has invaded, such as Italy and France (Malumphy et al. 2012; Garonna et al. 2015; OEPP/EPPO Reporting service, 2021). Therefore, there is growing interest in the potential use of *T. montezumae* in Europe as biocontrol agent in an integrated pest management programme.

The sequencing of the mitochondrial genome (mitogenome) of *T. montezumae* will be useful for several ways: 1) as a starting point for reconstructing the phylogenetic history of the genus *Thalassa*; 2) to correlate the migrations of this ladybird species with its hosts, and 3) to provide specific molecular markers useful for identifying spatial dispersal mechanisms in possible future experimental releases of this organism.

## Material and methods

Individual specimens (about 50) were collected in the Pine cay of the Turks and Caicos Islands (21°52′42″ N − 72°05′25″ W) and stored in 95% ethanol. Total DNA was extracted from a single individual using the QIAmp Mini Kit (Qiagen, Hilden, Germany) and the QIAcube automatic nucleic acid extractor (Qiagen, Hilden, Germany) according to the manufacturer’s recommended protocol. Amplification of the 5′ region of the COX 1 mitochondrial gene (‘barcode’ fragment) was performed according to Folmer et al. (1994) using primers LCO1490 5′-GGTCAACAAATCATAAAGATATTGG-3′ and HCO2198 5′-TAAACTTCAGGGTGACCAAAAAATCA-3′.

A 200-bp fragment library was generated by enzymatic digestion of total DNA using the Ion Xpress Plus Fragment Library Kit (Thermo Fisher) (Fig. S1). Next Generation Sequencing was performed on IonTorrent S5 System (Thermo Fisher) using Ion 510^™^ Chip (Thermo Fisher). Raw data were quality trimmed at a threshold level of 90%, removed adapter sequences and duplicate reads, and independently assembled using SPAdes v3.1.0 (Bankevich et al. 2012) and MITObim v.1.9.1 (Hahn et al. 2013).

The SPAdes assembly was obtained using three different k-mer settings (K_def_ [21, 33, 65], K_Short_ [21, 28, 33, 55, 65] and K_Long_ [21, 33, 55, 61, 71, 81, 91, 101, 111, 121]). The largest contig consensus sequence obtained from the *de novo* assembly of the mitogenome was identified through homology search using the mitochondrial COX I gene ‘barcode’ fragment as query using BANDAGE v. 0.8.1 (Wick et al. 2015). The MITObim assembly was obtained using the baiting algorithm with the barcode COX 1 gene fragment as the ‘bait’.

The contig sequence was annotated using MITOS2 (Bernt et al. 2013) and tRNAscan-SE 2.0 (Lowe and Eddy 1997). The mitogenome map of *T. montezumae* was generated using Geneious v. 2023.2.1 (Biomatters Ltd.) and the same software was used to generate circular %CG plot using a window size of 50 bp.

Sequences of protein-coding genes were extracted and concatenated from the annotated genome, and the codon usage table was calculated.

For phylogenetic analyses, 25 complete mitogenome sequences from 20 Coccinellidae species were downloaded from GenBank, as well as the mitogenome sequence of *Anoplophora glabripennis* (Motschulsky, 1853) as an outgroup. The 13 orthologous PCGs were aligned; the resulting alignments were concatenated with Geneious v. 2023.2.1. Neighbor joining phylogenetic tree was computed using Geneious v. 2023.2.1 considering the Tamura-Nei distance matrix with equal frequencies in transition and transversion rates and tested with 1000 Jackknife pseudoreplicates. Maximum likelihood phylogenetic tree was obtained using Mega 11 (Tamura et al. 2021) assuming the same substitution matrix and tested using 1000 bootstrap pseudoreplicates.

## Results and discussion

**Figure 2.**
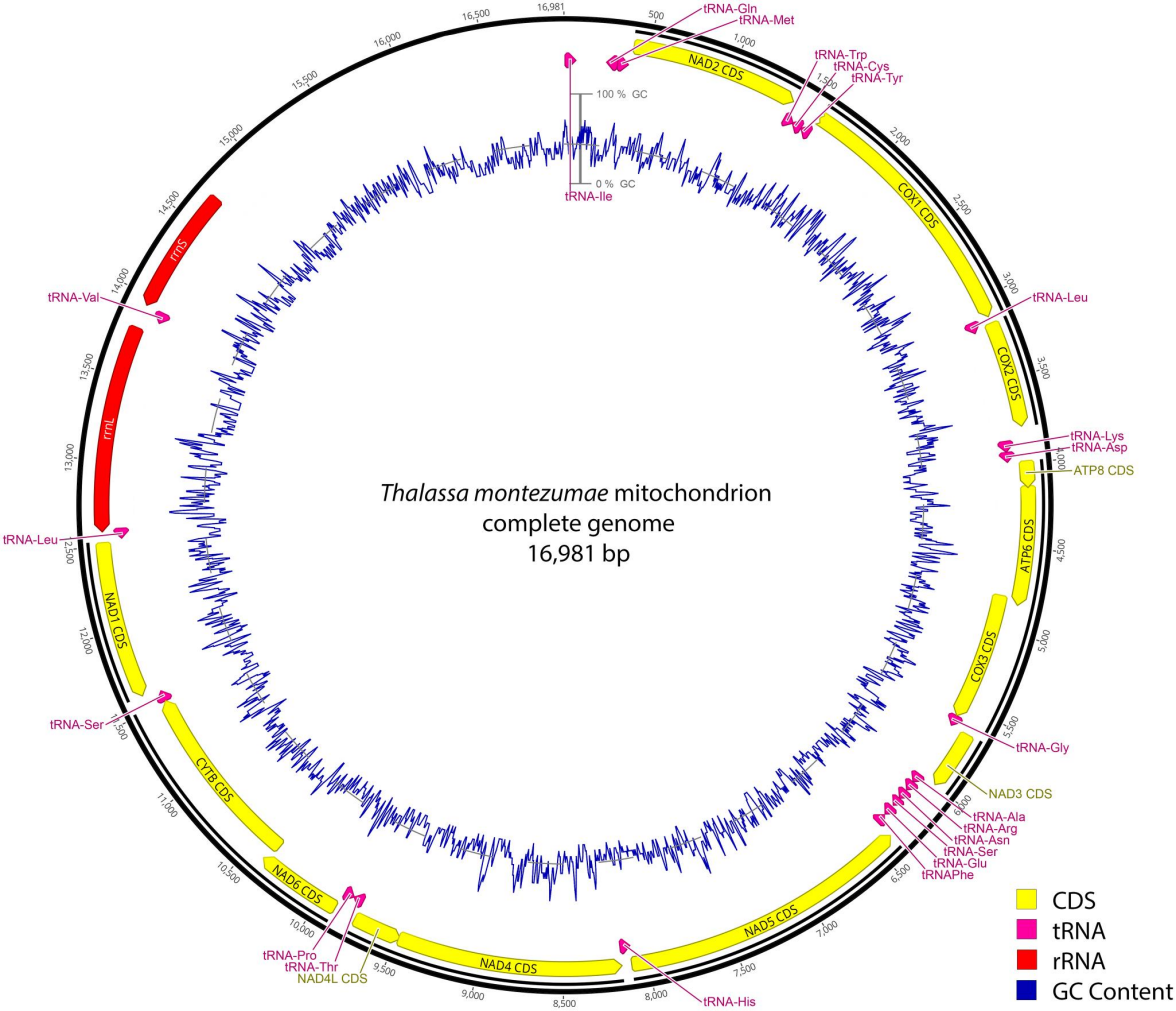
Gene arrangement in mitochondrial genome of *Thalassa montezumae* (Accession: PP865227). Visualization of MITOS2 and tRNAscan-SE 2.0 prediction using Geneious v. 2023.2.1.

Identical contig sequences were obtained with both SPAdes and MITObim assemblers. The mitogenome of *T. montezumae* was 16,981 bp long (Accession Number: PP865227) with an average coverage of 193 ± 83 (Supplementary Figure S1). It contains 13 protein-coding genes (PCGs), 22 transfer RNA (tRNA) gene, 2 ribosomal RNA (rRNA) genes and one control region. The overall nucleotide composition of the mitogenome showed a GC content of 22.8% and a base composition of 39.9% adenine, 37.3% thymine, 8.4% guanine, and 14.3% cytosine (Figure 2).

**Figure 3.**
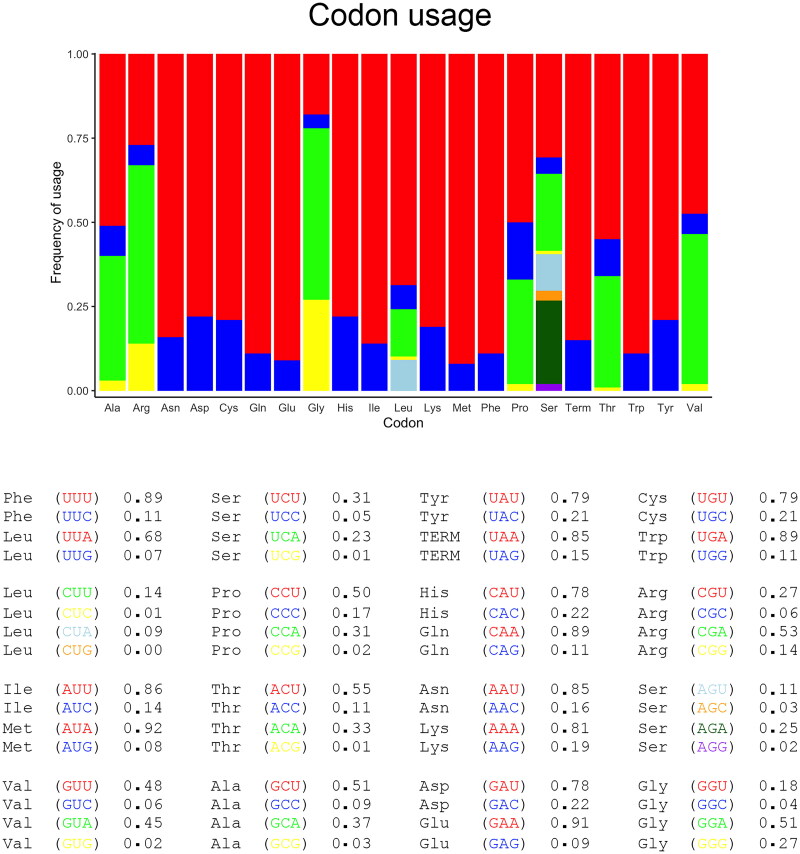
Codon usage in mitochondrial coding sequences of *Thalassa montezumae.*

Codon usage in protein coding genes showed a clear bias, in addition the most used codon often differed from the tRNA gene present in the mitochondrial DNA (Figure 3); this difference suggests a frequent use of wobbling in codon-anticodon coupling. There was no single preference in start codon usage (AUG 0.38, AUU 0.38, AUA 0.08, AUC 0.08, and TCG 0.08), although COX I start codon was atypical in Coleoptera (TCG) but common in Diptera (Beard et al. 1993). End codons showed a clear preference for UAA termination (0.85) over UAG (0.15).

**Figure 4.**
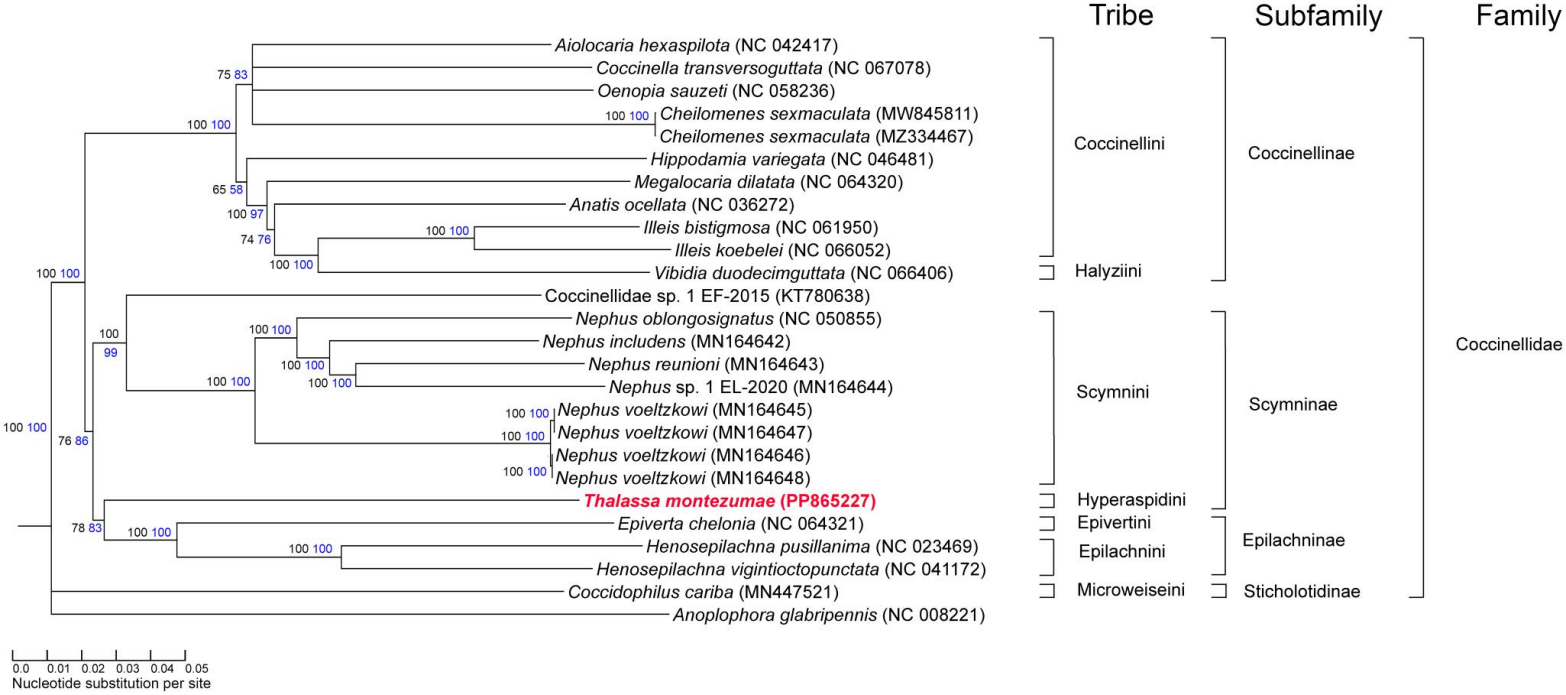
Phylogenetic tree of coccinellid species computed from 13 concatenated mitochondrial PCGs. Near the branches in black the neighbor joining jackknife values and in blue the maximum likelihood bootstrap values. The following sequences were used: NC042417 (Seo et al. 2019), NC067078 (NCBI Direct Submission), NC058236 (NCBI Direct Submission), MW845811 (unpublished), MZ334467 (Cui et al. 2021), NC046481 (Hao et al. 2019), NC064320 (NCBI Direct Submission), NC036272 (NCBI Direct Submission), NC061950 (Zhu et al. 2022), NC066052 (NCBI Direct Submission), NC066406 (Yan et al. 2020), KT780638 (NCBI Direct Submission), NC050855 (Magro et al. 2020), MN164642 – MN164648 (Magro et al. 2020), PP865227 (this work), NC064321 (Zhang et al. 2023), NC023469 (Behere et al. 2016), NC041172 (NCBI Direct Submission), MN447521 (Nattier and Salazar 2019), NC008221 (Fang et al. 2016).

Both the phylogenetic reconstruction algorithms computed a phylogenetic tree with the same topology (Figure 4). Although few complete and referenced Coccinellidae mitogenomes are publicly available, the phylogenetic reconstruction based on mitochondrial protein-coding genes respected the relationships previously defined by Seago et al. (2011). The Coccinellidae subfamily was supported by high jackknife and bootstrap values. More specifically, the monophyletic origin of the Coccinellinae subfamily was conserved in the reconstruction, but the paraphyletic nature of the Scymninae subfamily (Magro et al. 2010) could not be represented due to the complete absence of many taxa of this group.

## Supplementary Material

Supplementary Figure 1.tif

## Data Availability

A specimen was deposited at the entomological collection of CREA-DC (Firenze) (Contact person: Giuseppino Sabbatini, email: giuseppino.sabatini@crea.gov.it) under the voucher number CREADC-COLL-FI-482551A. DNA was deposited in the same location (Contact person: Agostino Strangi, email: agostino.strangi@crea.gov.it) under the voucher number CREADC-DNA-FI-482551A. All data obtained in this analysis are available in GenBank at the accession number PP865227. The associated BioProject, Bio-Sample numbers, and SRA are PRJNA1083940, SAMN40262580 and SRR29231871 respectively.
