# Supplementary figures and images for: The complete mitogenome and phylogenetic analysis of Thalassa montezumae Mulsant, 1850 (Coleoptera: coccinellidae)
Source: Mitochondrial DNA B Resour. 2024 Oct 7;9(10):1355–9. doi: 10.1080/23802359.2024.2412230 (PMC11459744; doi:10.1080/23802359.2024.2412230)

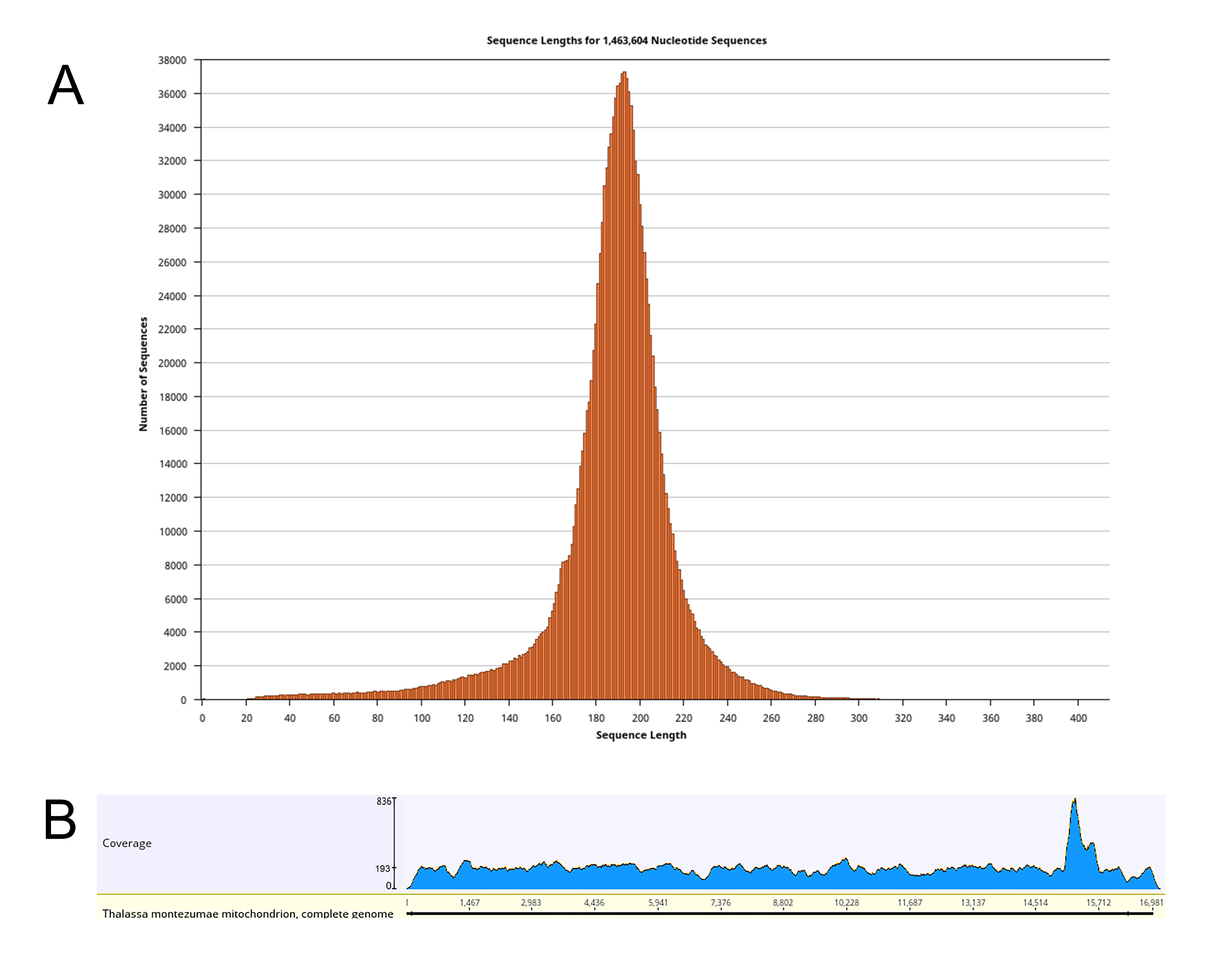

Supplement: Supplementary Figure 1.tif [file TMDN_A_2412230_SM1822.tif]
